# Microstructurally Informed Subject-Specific Parcellation of the Corpus Callosum using Axonal Water Fraction

**DOI:** 10.21203/rs.3.rs-3645723/v1

**Published:** 2023-11-25

**Authors:** Sohae Chung, Els Fieremans, Dmitry S. Novikov, Yvonne W. Lui

**Affiliations:** New York University Grossman School of Medicine; New York University Grossman School of Medicine; New York University Grossman School of Medicine; New York University Grossman School of Medicine

**Keywords:** corpus callosum, parcellation, axonal water fraction, diffusion MRI, microstructure

## Abstract

The corpus callosum (CC) is the most important interhemispheric white matter (WM) structure composed of several anatomically and functionally distinct WM tracts. Resolving these tracts is a challenge since the callosum appears relatively homogenous in conventional structural imaging. Commonly used callosal parcellation methods such as the Hofer/Frahm scheme rely on rigid geometric guidelines to separate the substructures that are limited to consider individual variation. Here we present a novel subject-specific and microstructurally-informed method for callosal parcellation based on axonal water fraction (ƒ) known as a diffusion metric reflective of axon caliber and density. We studied 30 healthy subjects from the Human Connectome Project (HCP) dataset with multi-shell diffusion MRI. The biophysical parameter ƒ was derived from compartment-specific WM modeling. Inflection points were identified where there were concavity changes in ƒ across the CC to delineate callosal subregions. We observed relatively higher ƒ in anterior and posterior areas consisting of a greater number of small diameter fibers and lower ƒ in posterior body areas of the CC consisting of a greater number of large diameter fibers. Based on degree of change in ƒ along the callosum, seven callosal subregions can be consistently delineated for each individual. We observe that ƒ can capture differences in underlying tissue microstructures and seven subregions can be identified across CC. Therefore, this method provides microstructurally informed callosal parcellation in a subject-specific way, allowing for more accurate analysis in the corpus callosum.

## INTRODUCTION

The corpus callosum (CC) is the largest commissural fiber bundle with more than 200 million axons, connecting left and right hemispheres of the brain. It is critical to interhemispheric communication and the global transfer of information across the brain. The CC is important in neurological disease and commonly involves conditions such as traumatic brain injury (Levin et al. 2000), demyelinating disorders (Evangelou et al. 2000; Ge et al. 2004) and brain tumor extension (Ho et al. 2013). Also, it has been implicated in a variety of learning, behavioral and affective disorders such as dyslexia (von Plessen et al. 2002), schizophrenia (Narr et al. 2002), depression (Lacerda et al. 2005) and autism (Prigge et al. 2013).

Commonly, the CC is divided into 3–5 arbitrary subregions from anterior to posterior to facilitate anatomic and imaging studies because different subregions connect specific cortical regions, serve different purposes, and have different propensities for disease. Unfortunately, there is a lack of clear boundaries that allow in vivo imaging parcellation of the CC since the structure appears relatively homogenous on conventional structural imaging. Most common research approaches rely on arbitrary, gross geometry to separate the CC into parts: for example, the Witelson scheme (Witelson 1989) defines subdivisions at 1/3, 1/2, 2/3, and 4/5 along the total callosal length anterior to posterior that is based on a mix of non-human primate and human datasets; Hofer and Frahm (Hofer and Frahm 2006), on the other hand, suggested subdivisions at 1/6, 1/2, 2/3, and 3/4 along the callosal length basing their system on presumed cortical projections derived from DTI-based fiber tractography (Basser and Pierpaoli 1996). However, such schemes ignore any individual variation in callosal structure which is clearly present even at a macroscopic level. Moreover, differences in fiber composition across the CC are observed on microscopy of human autopsy specimens, revealing mainly higher density of small and mid-diameter axons (< 2 μm in diameter) at anteriorly, large axons (> 2 μm in diameter) in the posterior mid-body, and mixed-size axons most posteriorly (Aboitiz et al. 1992).

Recent advances in compartment-specific white matter (WM) modeling of multi-shell diffusion MRI provide promising imaging markers that reflect underlying tissue microstructure (Fieremans et al. 2011; Novikov et al. 2018). In particular, axonal water fraction (ƒ) representing the volume of intra-axonal water relative to total intra and extra-axonal water volume, is known to reflect axon caliber and axon density based on animal and human studies with pathologic correlate (Jelescu et al. 2016; Margoni et al. 2019; Barazany et al. 2009). Distributions of varying axon caliber and density are observed in different sectors of the CC using electron microscopy (Aboitiz et al. 1992). Thus, we hypothesize that ƒ is able to capture differences in fiber composition as they vary along the anteroposterior extent of the midline CC. In this study, we present a novel subject-specific method for callosal parcellation based on the biophysical parameter ƒ and compare the results against callosal subregions as defined by the commonly used Hofer and Frahm scheme (Hofer and Frahm 2006).

## MATERIALS AND METHODS

### Subjects

This study includes 30 healthy subjects (age range, 22–35 years; 15 males) with multi-shell diffusion images from the Human Connectome Project (HCP) (Van Essen et al. 2013), an open-access multi-center dataset with high-quality 3T MR images. Institutional review board approval and participants’ informed consent were obtained at the participating institutions. Subjects have no documented history of mental illness, neurological disorder, or physical illness.

### Diffusion MRI Data

MRI data were acquired on two 3T Connectome scanners (Skyra, Siemens, Erlangen, Germany) with a 32-channel head coil. Diffusion imaging was performed with b-value = 1000, 2000, 3000 s/mm^2^ along 90 diffusion encoding directions for each, using multiband (factor of three) spin-echo EPI with 6/8 partial fourier. For this study, we used only b-values up to 2000 s/mm^2^ since high b-values are typically thought to distort quadratic fitting of diffusion kurtosis imaging (DKI) (Jensen et al. 2005). Other imaging parameters are: FOV = 210 mm × 210 mm, resolution = 1.25 × 1.25 × 1.25 mm^3^, matrix = 168 × 144, 111 slices, TR/TE = 5520/89.5 ms, bandwidth = 1488 Hz/pixel.

### Image Processing and Analyses

#### Diffusion MRI Processing

We used datasets that were preprocessed including b0 image intensity normalization, echo planar imaging distortion correction (FSL’s function, ‘topup’), eddy current and motion correction (FSL’s function, ‘eddy’), and gradient nonlinearity correction (Glasser et al. 2013).

Axonal water fraction, ƒ, is calculated by ƒ = Kmax/(Kmax+3), where Kmax is the maximum kurtosis overall diffusion directions (Fieremans et al. 2011) using in-house image processing software.

### Corpus Callosum Parcellation and Evaluation

**Figure 1:**
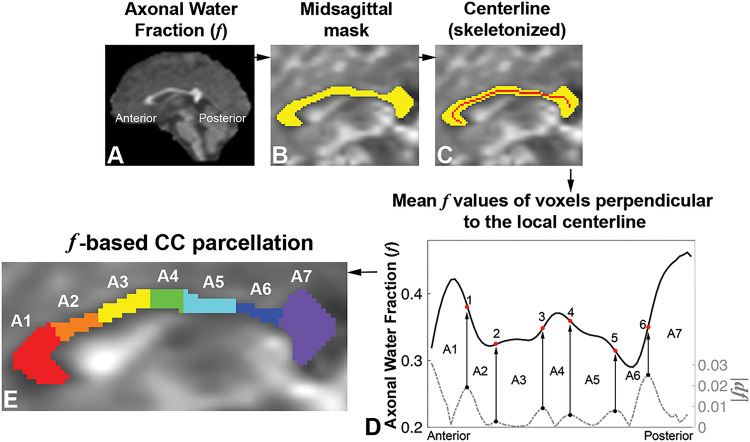
Procedure for axonal water fraction ƒ-based midsagittal corpus callosum (CC) parcellation, showing the ƒ map (A), callosal mask (yellow) overlaid on the b = 0 s/mm^2^ diffusion image (B), midsagittal callosal centerline (C; red line), and plot of mean ƒ values of the voxels perpendicular to the local centerline from anterior to posterior as fractional distance of callosal length and six inflection points (concavity changes along the plot; dƒ, 1^st^ derivative of the ƒ plot) indicating subdivisions where the composition of the microstructure changes between subregions (D). Seven callosal subregions (A1-A7) are present (E).

Parcellation of the CC followed the following procedure (Fig. 1): 1) obtaining callosal masks in the midsagittal plane using the JHU ICBM-DTI-81 WM labels atlas (Mori et al. 2008); 2) generating the callosal centerline by using FSL’s ‘tbss_skeleton’ commend (Lee et al. 1994); 3) plotting mean ƒ value of voxels perpendicular to the local centerline along the CC; and 4) identification of inflection points where there are concavity changes in ƒ (i.e., where the second derivative of ƒ = 0) to delineate callosal subregions based on the highest gradient in ƒ across the CC.

The results were compared with the Hofer and Frahm’s geometric partitioning scheme (Hofer and Frahm 2006) placing subdivisions at 1/6, 1/2, 2/3 and 3/4 along the callosal length for each subject. In addition, we employed whole-brain fiber tractography using MRtrix3 (Brain Research Institute, Melbourne, Australia) (Tournier et al. 2019) to map the cortical projections of the CC subregions derived from our method. Tracking parameters included 10 million streamlines with second-order integration over fiber orientation distributions (iFOD2), a step size of 0.6 mm, and a minimum length of track of 6.25 mm. Among whole-brain fiber tracts, only streamlines that traversed each callosal subregion were selected. The projected cortical regions were referred to by Brodmann area number (Brodmann 2006).

## RESULTS

**Figure 2:**
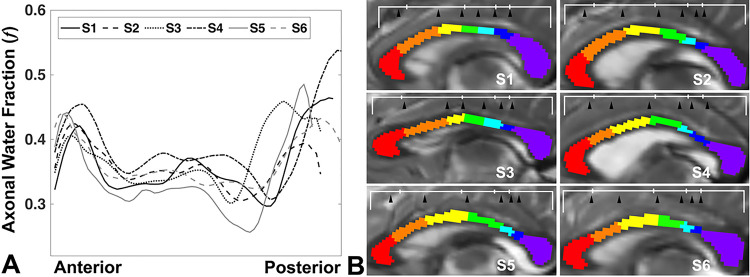
Plots of axonal water fraction ƒ across the corpus callosum in 6 representative subjects (A) plotted as fractional distance of callosal length. Substantial variation is present across individuals though the major contour features are preserved and, of interest, the number of partitions based on changes in ƒ concavity is the same in all subjects. Seven callosal subregions based on ƒ reflect microstructural properties such as axon density and size and are specific for each individual subject (B). The results (black arrowheads) show considerable individual variability not accounted for using methods based on fixed ratios (white bar; the Hofer and Frahm parcellation scheme). See Figure S1 for all 30 subjects.

Figure 2A shows that ƒ is relatively higher in anterior and posterior regions and lower in the posterior body region of the CC in representative subjects. Six inflection points of ƒ are consistently found leading to the delineation of seven subregions (Fig. 2B). On average, borders between subregions are identified at 1/8, 1/3, 1/2, 2/3, 5/7 and 4/5 of the total callosal length going from anterior to posterior. As shown in Fig. 2B, our borders (black arrowhead) do differ from Hofer and Frahm’s geometric borders (top white bar) for all individuals. The pattern of ƒ is consistent throughout all 30 healthy subjects and the value of ƒ ranges from 0.38 to 0.81 in the CC (Fig. S1).

**Figure 3:**
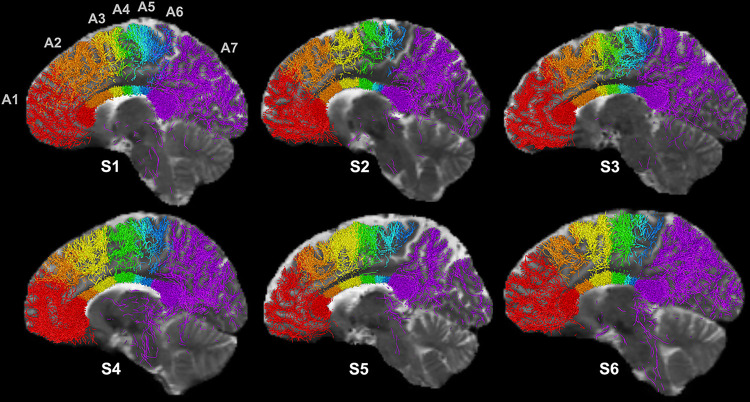
Cortical projections of callosal fiber bundles from the seven subregions identified are shown for the representative six subjects shown in Fig. 2. The cortical projections include prefrontal (red; Brodmann 10–11), frontal (orange; Brodmann 9), supplementary motor (yellow; Brodmann 8), premotor (green; Brodmann 6), motor (blue; Brodmann 4), sensory (navy; Brodmann 1–3), and parietal (purple; Brodmann 7) combined with temporal and occipital regions.

Figure 3 shows cortical projections of the callosal fiber bundles from the seven subregions, including prefrontal (A1: Brodmann 10–11), frontal (A2: Brodmann 9), supplementary motor (A3: Brodmann 8), premotor (A4: Brodmann 6), motor (A5: Brodmann 4), sensory (A6: Brodmann 1–3), and parietal (A7: Brodmann 7) combined with temporal and occipital regions, which are consistent with previous reports (Fabri et al. 2014; Park et al. 2008). The callosal parietal, temporal and occipital fiber bundles could not be separated.

## DISCUSSION

We present a novel callosal parcellation method using a biophysical parameter ƒ derived from advanced WM modeling based on multi-shell diffusion MRI. This study shows that ƒ does indeed capture differences in underlying tissue microstructures that are likely reflective of axon caliber and density across the CC ventrodorsally. We observe relatively higher ƒ in the anterior regions that contain about 72% of thinner, lightly myelinated fibers with small diameters of 0.2–1 μm in the total fiber population, and lower ƒ in the posterior mid-body regions that contain a great number of thicker, heavily myelinated fibers with relatively larger diameter (> 3 μm) as reported using electron microscopy by Aboitiz, et al (Aboitiz et al. 1992).

Current commonly used geometric parcellation schemes relying on fixed partitioning ratios such as the Witelson and Hofer/Frahm methods are not sensitive to individual variability of underlying tissue compositions. Our method is consistently able to separate WM bundles that track to either side of the marginal sulcus which separates the paracentral lobule from the precuneus, anatomically and functionally distinct areas. This would not always be expected to be the case using simple geometric parcellation methods across all individuals; for example, S5 (Fig. 2B) shows that the Hofer/Frahm scheme combines portions of the sensory sector (blue) and the precuneus (purple) while our method clearly separates them. In addition, our method is able to distinguish specific callosal subregions associated with frontal (A2: Brodmann 9), supplementary motor (A3: Brodmann 8) and premotor (A4: Brodmann 6) areas that are previously conglomerated in the Hofer/Frahm scheme. Interestingly, Fig. 3 shows the supplementary motor area (SMA) (A3; yellow) to be the most variable in terms of the cross-sectional area represented in the CC among these callosal regions. The SMA is known to be variable across individuals without good predictors of who may suffer SMA syndrome after frontal lobe surgery or insult (Ribas 2010; Baker et al. 2018). Being able to accurately parse cross-hemisphere tracts that contribute to specific anatomic and functional pathways could help guide future exploration such as the role of supplementary motor and premotor tracts in motor function and dysfunction.

Some limitations of the proposed method include being incumbent on adequate spatial resolution as is available in the HCP dataset, particularly in individuals who may have a narrow callosal isthmus (region between the body and splenium) which theoretically may lead to partial volume effects. In addition, the biophysical white matter modeling (Fieremans et al. 2011) used here to determine ƒ makes the assumption of highly aligned tracts. While this is probably a reasonable assumption in the CC as it is a structure with high transverse directionality, it is possible that some of the inability of the approach to resolve parietal, temporal and occipital white matter bundles separately within the posterior callosum is due to the presence of crossing fibers. Finally, this is a study of healthy adults; pathology of the CC may make parcellation difficult, though this in and of itself could serve as important information.

## CONCLUSIONS

We present a novel callosal parcellation method based on ƒ, reflective of biophysical factors of the underlying callosal microstructure. This method provides microstructurally-informed callosal subregions in a subject-specific way, allowing for more biologically-based analysis of regions of the CC.

## Data Availability

The datasets used in the current study are available in the HCP repository (https://www.humanconnectome.org/study/hcp-young-adult/data-releases).

## Abbreviations

CC: corpus callosum
Ƒ: axonal water fraction
HCP: Human Connectome Project
DKI: diffusion kurtosis Imaging
iFOD2: second-order integration over fiber orientation distributions
SMA: supplementary motor area
